# Varicella-Zoster Virus Vasculopathy: A Case Report Demonstrating Vasculitis using Black-Blood MRI

**DOI:** 10.4172/2155-9562.1000342

**Published:** 2015-12-28

**Authors:** Jay Shah, Husain Poonawala, Susan K Keay, Yafell Serulle, Andrew Steven, Dheeraj Gandhi, John W Cole

**Affiliations:** 1 Department of Neurology, University of Maryland School of Medicine, Baltimore, Maryland; 2 Department of Infectious Diseases, University of Maryland School of Medicine, Baltimore, Maryland; 3 Medical Service, VAMHCS, Baltimore, Maryland; 4 Department of Radiology, University of Maryland School of Medicine, Baltimore, Maryland; 5 Veterans Affairs Maryland Health Care System (VAMHCS) Geriatrics Research, Education, and Clinical Center, Baltimore, Maryland

**Keywords:** Stroke, Varicella zoster virus vasculopathy, Vasculitis, Zoster ophthalmicus, Black-blood MRI

## Abstract

Infections are rare but important causes of stroke. Among these, varicella zoster virus has been known to cause ischemic stroke. During an attack of herpes zoster ophthalmicus, it has been hypothesized that the virus replicates in the trigeminal ganglion and travels via the trigeminal nerve centrally to cause cerebral vasculopathy. Here we present a case of a 69 year-old Caucasian immunocompromised woman who suffered recurrent ischemic infarcts within the same vascular distribution following an episode of zoster ophthalmicus three months prior. An imaging technique termed black-blood magnetic resonance imaging was utilized to aid in the diagnosis of cerebral vasculitis. The case is used to provide a literature review of the pathogenesis, diagnosis, and treatment of cerebral varicella zoster vasculopathy. In situations where an isolated unilateral cerebral vasculopathy is identified, neurologists are urged to consider varicella zoster as a treatable etiologic agent, as untreated vasculopathy can lead to further strokes.

## Background

We describe a case of varicella zoster virus (VZV) vasculopathy in a 69 year old woman with myasthenia gravis on immunosuppressive therapy who presented with recurrent strokes in the same vascular territory three months after an episode of herpes zoster ophthalmicus. Initial imaging with magnetic resonance imaging (MRI) and magnetic resonance angiogram (MRA) could not distinguish between atherosclerosis and vasculitis. A black-blood magnetic resonance imaging (BB-MRI) demonstrated inflammation in the walls of the vascular territories responsible for the strokes. Isolation of VZV DNA from the cerebral spinal fluid (CSF) and demonstration of vasculitis on the BB-MRI confirmed the presence of VZV vasculopathy.

## Historical Evidence

Varicella zoster virus is a human alpha-herpesvirus that causes varicella (chickenpox) with primary infection. Subsequently, the virus becomes latent in cranial nerve and dorsal root ganglia along the neuraxis. As cell-mediated immunity wanes, the virus may reactivate to cause zoster which can lead to various complications, including vasculopathy [1]. While the literature on this entity is limited, VZV vasculopathy was initially described as herpes zoster ophthalmicus with contralateral hemiplegia in 1896 [2]. In a case report, Gilbert postulated that granulomatous angiitis described by Craviato and Feigin was likely due to VZV vasculopathy [2]. Linneman and Alvira were the first to demonstrate the presence of virus-like particles characteristic of herpes viruses in the vessel wall of a patient with granulomatous angiitis who died from disseminated herpes zoster [3]. Eidelberg et al. demonstrated the presence of VZV-specific antigens in the tunica media of cerebral blood vessels in two patients who died from strokes following herpes zoster [4]. Gilden et al demonstrated the presence of VZV antibody in the CSF and VZV DNA and VZV-specific antigen in the basilar, vertebral, anterior middle and posterior cerebral arteries of a patient with waxing and waning vasculitis [1], features that were seen in other patients with VZV vasculopathy [5,6].

Mackenzie et al. [7] described herpes zoster ophthalmicus with hemiplegia in four patients and demonstrated focal stenosis on cerebral angiography ipsilateral to the herpes zoster with normal angiographic features contralaterally. Crucially, the authors also suggested that the infection spread to the vessel wall via sensory nerve fibers innervating the intracranial potion of the internal carotid arteries that originated from the ophthalmic division of the trigeminal nerve, a neural pathway that was demonstrated in a cat model [8]. Berkefeld et al. described a patient with right hemiparesis with a positive VZV serum IgG and positive serum VZV DNA in whom they were able to demonstrate vessel wall enhancement in the affected vascular territory that resolved with acyclovir and steroids [9].

## Case Presentation

A 69 year-old woman with a history of myasthenia gravis on immunosuppressant therapy (mycophenolate 1000 mg BID and prednisone 5 mg QD) for the previous two years presented to our facility with acute left-sided weakness and numbness. She reported two recent ischemic stroke hospitalizations over the preceding 3 months and that she was fully compliant with her ongoing post-stroke medical management. Initial non-contrast computed tomography (CT) of the head was negative for hemorrhage or evidence of acute ischemia, but did demonstrate regions consistent with her prior infarcts in the right fronto-parietal regions. Intravenous thrombolytic therapy was not administered secondary to timing and improving symptoms. National Institutes of Health Stroke Scale (NIHSS) score was 8 with left-sided hemiparesis and paresthesias involving face, arm, and leg.

Further review of her prior history revealed that approximately 3 months before her current presentation she had experienced acute onset of left lower extremity weakness with imaging demonstrating scattered ischemic infarcts in the right frontal lobe. At that time, aspirin and statin therapy was initiated and she was transferred to a rehabilitation facility. Two weeks later, while at the rehabilitation facility, she experienced acute left facial droop and left upper extremity weakness with no sensory loss or neglect with imaging demonstrating new scattered ischemic infarcts involving the right fronto-parietal region and corona radiata. Computed tomography angiogram (CTA) of the head demonstrated marked irregularity of the right A1 segment of the anterior cerebral artery (ACA). There was also calcification in various bilateral cerebral vessels without flow-limiting stenosis. Intracranial atherosclerosis was hypothesized as the likely culprit of her recurrent strokes and dual anti-platelet therapy with aspirin and clopidogrel was initiated; she continued on her statin. After a short course of rehabilitation she was discharged home. She then presented to our facility approximately three months after the initial stroke.

**Figure 1:**
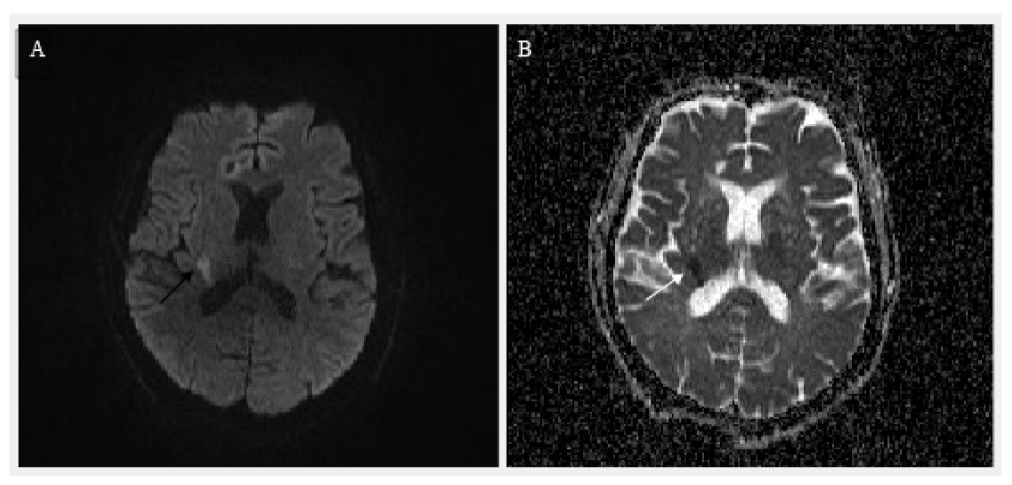
MRI of the brain demonstrating acute infarct. (A) MRI demonstrates hyperintensity within the right internal capsule on diffusion weighted images (black arrow). (B) Acute infarction is confirmed by hypointensity within the same territory on apparent diffusion coefficient (white arrow).

**Figure 2:**
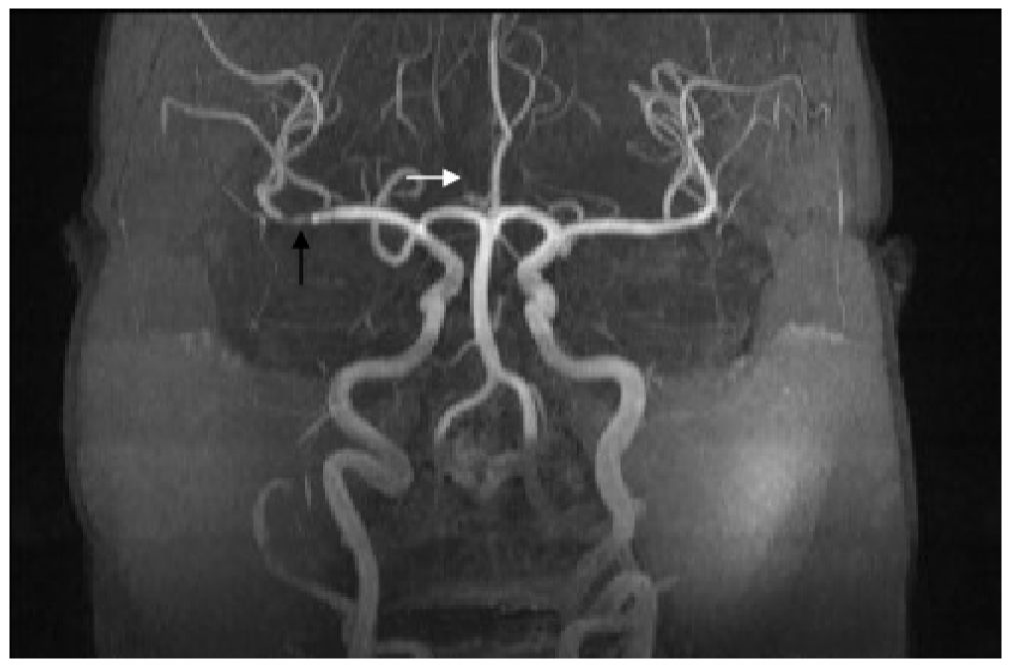
MR Angiogram of the head and neck. MRA demonstrates stenosis of M1 segment (black arrow) of right MCA and absence of right ACA (white arrow points to normal location).

During her hospitalization in our facility, work-up revealed a hemoglobin A1C of 4.7% and a LDL of 39 mg/dL. Transthoracic echocardiogram showed a preserved ejection fraction with moderate calcified aortic stenosis, no patent foramen ovale (PFO), and no atrial thrombus. EKG and telemetry demonstrated normal sinus rhythm. MRI demonstrated new acute scattered ischemic infarctions involving the right corona radiata and right posterior limb of internal capsule (Figure 1). 3D Time-of-flight non-contrasted magnetic resonance angiogram (MRA) re-demonstrated the right ACA stenosis with additional stenosis of right M1 segment of middle cerebral artery (Figure 2) that was not apparent on the previous CTA from two months prior. Upon further questioning, the patient stated that she had shingles approximately 3 months prior to her initial stroke event that involved the right side of her face (forehead - V1 distribution) and eye. At that time she was treated with oral anti-viral therapy. Speculation of a cerebral VZV vasculopathy was raised given the likely history of a herpes zoster ophthalmicus infection six months earlier followed by three stroke events despite escalating medical management with dual anti-platelet therapy and high-dose statin therapy. Further, the strokes were all in the same vascular territory, with corresponding and progressive stenotic lesions seen on MRA. While VZV vasculopathy seemed likely, it remained inconclusive whether the strokes were due to an ongoing VZV vasculopathy or pre-existing atherosclerosis.

**Figure 3:**
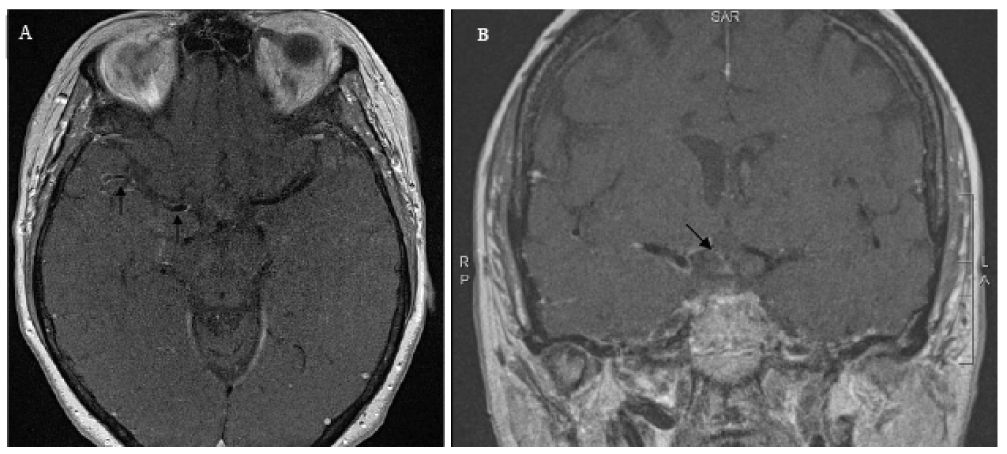
Black-blood MRI demonstrates unilateral enhancement. (A) Post-contrast black blood MRI images demonstrate enhancement of the right MCA (black arrows) and (B) of the right ACA (black arrow).

To differentiate between vasculitis and atherosclerosis causing stenosis, intracranial arterial wall imaging (black blood MRI) was performed using a 3T MR scanner. The arterial wall imaging protocol consisted of pre- and post-contrasted axial and coronal T1-weighted images with inversion recovery, BB-MRI demonstrated circumferential wall enhancement of the supraclinoid portion of right internal carotid artery, A1 and proximal A2 segments of the right ACA, and M1 and proximal M2 segments of the right MCA, compatible with vasculitis (Figure 3). Subsequent analysis of the cerebral-spinal fluid (CSF) demonstrated 20 WBC/ml with lymphocytic pleocytosis with normal protein and glucose levels. CSF antibodies for lymphocytic choriomeningitis, measles, mumps, West Nile virus, and herpes simplex virus were negative. CSF antibodies for VZV were positive but there was no evidence of IgM antibody. PCR for the virus in CSF was positive revealing 4500 copies/milliliter.

The presence of VZV DNA in CSF and the pattern of vessel wall enhancement on the BB-MRI supported the clinical diagnosis of VSV vasculopathy, as such a digital subtraction angiogram (DSA) was not deemed necessary. With guidance from our Infectious Disease Consult Service, the patient was started on treatment with intravenous acyclovir (10 mg/kg in three daily doses) for total of 2 weeks, then switched to valacyclovir 500 mg BID as suppressive therapy in light of her continued MG immunosuppression therapy requirements. Following inpatient hospitalization, the patient was transferred to acute stroke rehabilitation and is now followed in our outpatient stroke clinic. At her most recent evaluation approximately 5 months after discharge, she reported no new neurological symptoms and improving residual deficits; her most recent examination was positive for left upper extremity pronator drift, 4/5 strength in left lower extremity, and diminished pain and temperature on the left side. She is ambulating with use of a rolling walker.

### Epidemiology

Herpes zoster has been shown to be associated with an increased incidence of stroke in large population-based studies [10-13]. Sreenivasan et al. demonstrated not only an increased short-term risk of stroke following zoster but also a long-term risk, especially when zoster developed at age <40 years [10]. Breur et al. were able to show an increased risk for transient ischemic attack (TIA) and myocardial infarction, but not stroke [14]. Stroke risk is higher with herpes zoster ophthalmicus [11-13] than with herpes zoster involving other dermatomes. The risk of stroke is highest immediately following an episode of zoster with subsequent time-dependent decline in incidence, but has been shown to be an independent risk factor for stroke up to 24 years after an episode of zoster [14]. In a cohort of 70 consecutive children with acute strokes [15], nearly 1/3rd of affected children had varicella in the preceding year, three-times greater than typical population norms. The risk of stroke in children with preceding varicella was highest in the first two months after the infection, with a subsequent time-dependent decline in incidence. Children with preceding varicella were more likely to present with hemiparesis and have recurrent vascular events.

Langan et al. demonstrated that patients treated for herpes zoster had a lower incidence of stroke than untreated patients [11]. Interestingly, patients with herpes zoster ophthalmicus had a >5 fold increase in stroke risk between weeks 5-12, with those receiving treatment reducing this risk by nearly half [11]. However, in another study of herpes zoster ophthalmicus, Lin et al. were unable to show a difference in stroke incidence among those treated and untreated [13].

### Clinical manifestations and diagnosis

The clinical features of VZV vasculopathy have been described as protean [16]. Patients classically present with hemiplegic stroke, but symptoms vary depending on which vascular territory is affected. Monocular vision loss due to occlusion of central retinal artery and posterior ciliary artery has been described [17]. Patients may have acute or chronic symptoms, with some presenting only with persistent headache or changes in mental status [17]. Case reports of varicella zoster vasculopathy causing venous sinus thrombosis, extra-cranial vasculitis, cranial neuropathies, spinal-cord infarction, aneurysm, subarachnoid hemorrhage, intracerebral hemorrhage, ectasia and dissection, peripheral arterial disease, giant cell arteritis, and retinal necrosis have been summarized [18].

In a review of 30 patients with confirmed VZV vasculopathy, 67% had CSF pleocytosis and 97% patients had abnormal findings on brain imaging, usually at the gray-white matter junction [19]. Only 13% had isolated large artery disease, 37% had isolated small artery involvement and 50% had mixed small and large vessel involvement. Angiographic evaluation showed vessel wall abnormalities in 70% of patients. Among the 11 immunocompromised patients, 6 (54%) had VZV DNA in their CSF compared to 3 of 19 (16%) immunocompetent patients. In the 11 immunocompromised patients, all 11 (100%) had anti-VZV IgG antibody in their CSF compared to 17 of 19 (89%) of the immunocompetent patients. Overall, the presence of VZV-specific IgG in the CSF was demonstrated in 28/30 patients, thereby confirming that CSF VZV-specific IgG is more sensitive than CSF VZV DNA detection to make a diagnosis of VZV vasculopathy [20].

### Pathogenesis

Varicella virus is the only known human virus to have been isolated from vessels responsible for cerebral ischemia or infarction [21]. Vasculopathy is initiated by transaxonal spread of virus from the trigeminal ganglion to the tunica adventitia, followed by replication and transmural migration to the tunica media [22]. However, evidence also exists for hematogenous spread of virus in VZV vasculopathy [23]. Autopsy studies have demonstrated the presence of VZV antigen and neutrophilic infiltrate in the adventitia in early infection [22,24]. During both early and late disease CD4+ and CD8+ T cells, macrophages and B cells are present in the adventitia and intima [24]. The tunica media in late VZV vasculopathy is characterized by the presence of VZV antigen but absence of inflammatory cells, suggesting an immunoprivileged territory. The involvement of vasa vasourum in early VZV vasculopathy may reflect virus induced vessel-wall remodeling [24].

Vascular remodeling occurs with the development of a neo-intima composed of smooth muscle cells that may have originated from the tunica media [22], a process also seen in the development of atherosclerotic plaque [25]. The changes in the vascular wall due to viral reactivation may lead to changes in arterial caliber and contractility that increase predisposition to stroke [24]. Transient autoantibodies to phospholipids and coagulation proteins (especially to Protein S with corresponding decrease in protein S concentration) have been described following acute varicella infection in children [26-30] and adults [31,32]; however, it is unknown if autoantibodies play a role in stroke or vasculitis following herpes zoster.

It is important to note that the immunosuppressed patient described in this case report developed VZV vasculopathy despite having been treated with oral antivirals when diagnosed with herpes zoster ophthalmicus. This suggests that host immunity may also play a role in determining the progression from herpes zoster to VZV vasculopathy. Further, it remains unclear from population based studies of stroke post herpes zoster and isolated cases of VZV vasculopathy if similar pathophysiological mechanisms are involved in both processes or if they represent a range of infection-triggered inflammatory response that is modulated by host immunity.

### Workup and treatment

As described above, imaging studies using BB-MRI and CSF evaluations for VZV-specific IgG and VZV DNA were central to our diagnosis. It is important to note that not all cases of VZV vasculopathy demonstrate angiographic abnormalities using traditional angiographic imaging techniques; moreover, if present, vasculopathy may also be difficult to discern from ongoing atherosclerotic disease. Nagel et al. document that 30% of patients with VZV vasculopathy do not have angiographic findings on MRI or CT [19]. Obusez et al. showed that BB-MRI was able to differentiate between vasculitis and reversible cerebral vasoconstriction [32]. In a study of six patients with proven VZV vasculopathy Cheng-Ching et al. use high-resolution MRI to demonstrate improvement in angiographic features following treatment [33]. However, both of these studies were performed retrospectively.

There are no randomized studies defining or comparing guidelines for the treatment of varicella zoster vasculopathy in immunocompromised or immunocompetent patients. In one study of 30 patients with VZV vasculopathy, 15 patients were treated with acyclovir alone of which 9 improved, 1 stabilized, and 5 worsened (3 of which improved with steroids). Of the 12 patients treated with steroids and acyclovir, 8 improved, 1 remained stable and 3 worsened [19].

The roles of antiviral drugs or VZV vaccines in stroke risk reduction from either primary or reactivated varicella virus infection are unknown. The optimal therapy for treatment of VZV vasculopathy for either immunocompetent or immunocompromised patients is also unknown and requires further research. Shown in Table 1 [4,19,34-42] is a summary of treatments and outcomes for adult immunocompromised patients with VZV vasculopathy reported in the literature. As indicated, 15 of the 30 (50%) of the patients received antiviral therapy alone, 12 of the 30 (40%) received both antiviral and corticosteroid therapy (the latter treatment was given either initially with the antiviral therapy, or was added subsequently to the antivirals), 1 (3%) received corticosteroids alone, and 2 (7%) received no antiviral or steroid therapy. Of the patients who were treated with antivirals alone, 12 of them (80%) stabilized or improved, and of the patients who were treated with antivirals and corticosteroids 9 of them (75%) also stabilized or improved; the one patient who was treated with corticosteroids alone also improved, but the 2 patients who received neither antiviral nor steroid therapy both died. Although randomized trials are lacking, these data suggest that antiviral therapy +/− corticosteroids should be given as initial therapy to all immunocompromised patients with VZV vasculopathy, and for those patients treated with antivirals alone, the addition of corticosteroids should be considered if they do not improve clinically on antivirals alone. Finally, as several patients including ours have recently been reported to have VZV vasculopathy associated with the use of newer, more potent immunosuppressive drugs, it is possible that a reduction of these types of immunosuppressive therapy (other than corticosteroids) to the extent possible at the time of diagnosis with herpes zoster may help to retard the progression to VZV vasculopathy and/or improve healing and clinical outcome [43-46].

## Conclusion

VZV vasculopathy should be considered in the setting of recent shingles infections, particularly among those with eye involvement and/or among patients that are immunocompromised. As described in our case, we were able to document isolated vessel wall inflammation through the use of BB-MRI that was not seen on traditional MRA, and that enhanced atypically as compared with atherosclerotic disease. We believe our case is among the first in the literature utilizing this imaging technique for real-time clinical decision-making to demonstrate vessel wall inflammation in a patient with VZV vasculopathy. As such, we propose BB-MRI as a methodology to screen patients presenting with acute stroke in the setting of a recent herpes zoster infection, as such patients would benefit from treatment with antiviral therapy. Combined with the detection of VZV IgG and/or VZV DNA from the CSF, this technique may improve the identification of patients with VZV vasculopathy and possibly demonstrate response to therapy. Further studies implementing BBMRI may also determine what percentage of strokes following herpes zoster are due to vasculitis and provide further insight into the pathophysiology of VZV vasculopathy. A review of the literature also indicates that antiviral +/− corticosteroid therapy results in 75-80% stabilization or improvement in neurologic findings in immunocompromised adults with VZV vasculopathy.

## Figures and Tables

**Table 1:** Reported treatments for VZV vasculopathy in immunocompromised patients

Reference	Number of patients	Underlying Illness	Antiviral Rx	Other Rx	Outcome
(34) JAMA Neurol 39: 785.	1	Hodgkin’s lymphoma	None	Prednisone 65 mg qd Cyclophosphamide (Total duration of all treatment said to be 7 months)	Improved mental status and seizures, but residual paraplegia
[4] Ann Neurol 19: 7-14.	2	Hodgkin’s lymphoma and diffuse histiocytic lymphoma	None	None	Both patients died
[35] Biomed Pharmacother 51: 449-54.	2	HIV infection	Acyclovir 30 mg/kg qd IV for 1 month	Corticosteroids for 1 month	Hemiparesis improved in both; patient with retinal necrosis had persistent loss of vision
[36]Dermatology 200: 173-175.	1	large B cell lymphoma	Acyclovir IV 10 mg/kg TID for 7 days	None	Improved
[37] IDSA 35: 330-333.	1	HIV infection	Acyclovir 10 mg/kg TID IV for 24 days, followed by Valacyclovir 2 GM po TID for 4 weeks	None	Improved
[38] NEJM 347: 1500-1503.	1	chronic lymphocytic leukemia	Acyclovir 10-15 mg/kg TID IV for 7 days	None	Improved
[39] Pathology 34: 88-93.	1	HIV infection	Acyclovir IV	None	Died
[19] Neurology 70: 853-60.	3	HIV infection (2 patients) and leukemia (1 patient)	Acyclovir IV (dose and duration not specified)	None	2 improved; 1 died
[19] Neurology 70: 853-60.	1	HIV infection	Acyclovir IV (dose and duration not specified) followed by Famvir 500 mg po TID (duration unknown)	None	Stabilized
[19] Neurology 70: 853-60.	1	HIV infection	Acyclovir IV (dose and duration not specified) followed by Valacyclovir for 4 weeks (dose unknown)	None	Improved
[19] Neurology 70: 853-60.	3	Lymphoma, “low CD4” of unknown etiology, and chronic lymphocytic leukemia	Acyclovir IV (dose and duration not specified)	Corticosteroids (po and/or IV)	2 progressed; 1 improved
[19] Neurology 70: 853-60.	3	Rheumatoid arthritis and systemic lupus erythematosus (1 patient with both), CREST syndrome, and HIV infection	Acyclovir IV (dose and duration not specified)	Later addition of corticosteroids (plus one patient received acetylsalicylic acid)	2 improved; 1 stabilized after addition of corticosteroids
[40] Rev Neurol 164: 61-71.	3	Osteosarcoma, heart/lung transplant, and systemic lupus erythematosus	Acyclovir 30 mg/kg qd IV (duration not specified)	None	Improved
[41] CID 48: 372-373.	1	HIV infection	Acyclovir IV (dose and duration not specified)	Corticosteroids	Died
[42] J Int Assoc Physicians AIDS Care 10: 144-145.	1	HIV infection	Acyclovir 10 mg/kg TID IV for 10 days	None	Improved

## Abbreviations

VZV: Varicella Zoster Virus
MRI: Magnetic Resonance Imaging
MRA: Magnetic Resonance Angiogram
BB-MRI: Black Blood Magnetic Resonance Imaging
CSF: Cerebrospinal Fluid
MCA: Middle Cerebral Artery
ACA: Anterior Cerebral Artery
